# Efficacy and safety of hybutimibe in combination with atorvastatin for treatment of hypercholesteremia among patients with atherosclerotic cardiovascular disease risk equivalent: A multicenter, randomized, double-blinded phase III study

**DOI:** 10.3389/fcvm.2022.888604

**Published:** 2022-08-16

**Authors:** Litong Qi, Jiyan Chen, Xiaodong Li, Xiaoyong Qi, Chunhua Ding, Xiaoping Chen, Xiang Gu, Wenliang Xiao, Shuiping Zhao, Yugang Dong, Mingqi Zheng, Kai Huang, Liangqiu Tang, Xiaomei Guo, Fang Wang, Guosheng Fu, Junxia Li, Yong Huo

**Affiliations:** ^1^Department of Cardiology, The First Hospital of Peking University, Beijing, China; ^2^Department of Cardiology, Guangdong Provincial People's Hospital, Guangdong Academy of Medical Sciences, Guangzhou, China; ^3^Department of Cardiology, Shengjing Hospital of China Medical University, Shenyang, China; ^4^Department of Cardiology, Hebei General Hospital, Shijiazhuang, China; ^5^Department of Cardiology, Aerospace Central Hospital, Beijing, China; ^6^Department of Cardiology, Western China Hospital of Sichuan University, Chengdu, China; ^7^Cardiology Department, Northern Jiangsu People's Hospital, Yangzhou, China; ^8^Department of Cardiology, The Third Hospital of Hebei Medical University, Shijiazhuang, China; ^9^Cardiovascular Department, The Second Xiangya Hospital of Central South University, Changsha, China; ^10^Department of Cardiology, The First Affiliated Hospital, Sun Yat-sen University, Guangzhou, China; ^11^Department of Cardiology, The First Hospital of Hebei Medical University, Shijiazhuang, China; ^12^Deparrtment of Cardiology, Xiehe Hospital, Tongji Medical College, Huazhong University of Science and Technology, Wuhan, China; ^13^Department of Cardiology, Yue Bei People's Hospital, Shaoguan, China; ^14^Department of Cardiology, Tongji Medical College, Tongji Hospital, Huazhong University of Science and Technology, Wuhan, China; ^15^Department of Cardiology, Beijing Hospital, Beijing, China; ^16^Key Laboratory of Cardiovascular Intervention and Regenerative Medicine of Zhejiang Province, Department of Cardiology, Sir Run Run Shaw Hospital, School of Medicine, Zhejiang University, Hangzhou, China; ^17^Senior Department of Cardiology, The Seventh Medical Center of PLA General Hospital, Beijing, China

**Keywords:** atherosclerotic cardiovascular disease risk equivalent, lipid profile, hybutimibe, atorvastatin, randomized controlled trial, cholesterol-absorption inhibitor

## Abstract

**Background:**

To evaluate the safety and efficacy of hybutimibe plus atorvastatin for lipid control in hypercholesterolemia patients with atherosclerotic cardiovascular disease risk equivalent.

**Methods:**

In this double-blind phase III study, we 1:1 randomly assigned 255 hypercholesterolemia patients with atherosclerotic cardiovascular disease to receive hybutimibe plus atorvastatin or placebo plus atorvastatin. The primary endpoint was the rate of change of plasma low-density lipoprotein-cholesterol (LDL-C) level at 12 weeks from baseline. The secondary endpoints were plasma total cholesterol (TC), triglyceride (TG), high-density lipoprotein-cholesterol (HDL-C), non-HDL-C, apoprotein (Apo) B, and 2-, 4-, 8-, and 12-week Apo A1 levels change rate and rates of change of plasma LDL-C levels at 2, 4, and 8 weeks from baseline.

**Results:**

From April 2016 to January 2018, 128 in the hybutimibe plus atorvastatin group and 125 in the atorvastatin group were included in modified intention-to-treat (mITT) analysis. After 12 weeks of treatment, LDL-C level changed from 2.61 mmol/L (±0.30) at baseline to 2.18 mmol/L (±0.45) in the hybutimibe plus atorvastatin group and from 2.58 (±0.31) mmol/L to 2.40 (± 0.46) mmol/L in the atorvastatin group (*P* < 0.0001), in mITT. The change rate in the hybutimibe plus atorvastatin group was significantly higher than that in the atorvastatin group (*P* < 0.0001); the estimated mean rates of change were −16.39 (95% confidence interval: −19.04, −13.74) and −6.75 (−9.48, −4.02), respectively. Consistently, in per-protocol set (PPS) analysis, the rate of change of LDL-C in the hybutimibe plus atorvastatin group was significantly higher than that in atorvastatin group. Significant decreases in the change rates of non-HDL-C, TC, and Apo B at 2, 4, 8, and 12 weeks (all *P* < 0.05) were observed for hybutimibe plus atorvastatin, while the differences were not significant for HDL-C, TG, and Apo-A1 (all *P* > 0.05). During the study period, no additional side effects were reported.

**Conclusions:**

Hybutimibe combined with atorvastatin resulted in significant improvements in LDL-C, non-HDL-C, TC, and Apo B compared with atorvastatin alone. The safety and tolerability were also acceptable, although additional benefits of hybutimibe plus atorvastatin were not observed compared with atorvastatin alone in HDL-C, TG, and Apo-A1.

## Introduction

The DYS Lipidemia International Study China (DYSIS-China) in 2012 based on the data from 122 hospitals covering 22 counties found that most patients receiving lipid-lowering therapy in China were at high or very high risk of atherosclerotic cardiovascular diseases (1, 2). Over the last 10 years, blood lipid control in China has substantially improved and the overall low-density lipoprotein-cholesterol (LDL-C) control rate increased from 8.9% in 2007 to 61.5% in 2012 (3, 4); however, the control of blood lipids in very high-risk patients is still poor. Most patients use statin monotherapy, since statin is the first-line therapy for lipid-lowering (5, 6). However, the average difference between LDL-C levels after treatment and target LDL-C levels in high-risk/extremely high-risk patients is still >30 mg/dl (0.777 mmol/l), indicating the lack of efficiency of statin monotherapy (7). Notably, patients using moderate-intensity statins had the highest LDL-C and non-high-density lipoprotein-cholesterol (non-HDL-C) compliance rates, while the use of high-intensity statin had the lowest LDL-C compliance rate. This is consistent with the conclusion of the Intensive Lipid-lowering Intervention Study for Patients with Acute Coronary Syndrome in China (CHILLAS): high-intensity statin does not enable Chinese patients to obtain additional lipid-lowering benefits, and moderate-intensity statin lipid-lowering therapy is suitable for Chinese patients (8, 9). Moreover, for Chinese patients, especially those at high or extremely high risk, combined lipid-lowering therapy might be a new beneficial option.

Hybutimibe, a new cholesterol-absorption inhibitor, exhibits a mechanism of action similar to that of ezetimibe to decrease lipids (10–12). Briefly, hybutimibe inhibits the absorption of cholesterol from food and bile acid, reduces the transport of cholesterol in the small intestine to the liver, limits the storage of cholesterol in the liver, and increases the clearance of cholesterol from the blood (11, 12). Hybutimibe is a new selective cholesterol-absorption inhibitor approved in China. In terms of its difference from ezetimibe, the hydroxyl group in its structure makes binding to glucuronic acid easier, which improves the rate of conversion in the form of glucuronidation (12, 13). Additionally, the overall clearance rate of metabolites of hybutimibe is higher, and the liver burden of patients was reported to be greatly reduced (13, 14). Hybutimibe has completed phase I and II studies in the U.S. and China. The results of the Chinese phase II study showed that, after 8 weeks of treatment, compared with placebo, hybutimibe significantly reduced LDL-C levels and was well-tolerated, whereas its efficacy was comparable with that of ezetimibe (11, 13).

To further evaluate the safety and efficacy of hybutimibe, a randomized, double-blind, multicenter phase III study was designed in hypercholesterolemia patients with atherosclerotic cardiovascular disease and/or “equal-risk diseases” who had not achieved the target blood LDL-C level after being treated with 10 mg of atorvastatin for at least 6 weeks.

## Materials and methods

### Study design and patients

This was a multicenter, randomized, double-blinded, double-dummy, placebo-, and positive-controlled phase III study to evaluate the efficacy and safety of hybutimibe in combination with atorvastatin for treatment of hypercholesteremia among those also suffering atherosclerotic cardiovascular disease and/or “equal-risk diseases.” This study was registered at ClinicalTrials.gov (NCT03433196). From April 2016 to January 2018, a total of 736 patients from 23 centers in China were screened; finally, 255 patients completed the screening lead-in period for dietary control, received atorvastatin at 10 mg/day, and were enrolled in this study.

The patients meeting the following inclusion criteria were included: aged 18–75 years; dietary control for more than 14 weeks; use of atorvastatin monotherapy at 10 mg/qd for at least 6 weeks; serum LDL-C level ≥2.07 mmol/L and ≤3.36 mmol/L (this target lipid blood levels was defined according to the target value of lipid-lowering therapy in very high-risk and intermediate-risk patients from Guidelines for the prevention and treatment of dyslipidemia in Chinese adults in 2007) (15), with two LDL-C measurements (time interval ≥1 week) during the lead-in period being within this range and the difference between the two LDL-C measurements being <12%; and etc. Patients were excluded if they had: homozygous familial hypercholesterolemia; severe organ disease; unstable atherosclerotic cardiovascular disease; drugs including fibric acid, probucol, warfarin, systemic steroids, cyclosporine, or other immunosuppressive agents within 12 weeks before enrollment; dietary evaluation score >5, and etc. For details, please refer to the information on ClinicalTrials.gov (NCT03433196).

All patients provided written informed consent before enrollment. The protocol of this study was approved by Peking University First Hospital institutional review board [No. (2015) Drug Registration No. (56)] and this study was conducted in accordance with the Declaration of Helsinki and Good Clinical Practice guidelines.

### Randomization, blinding, and treatment

After at least 6 weeks of screening, eligible patients with hypercholesterolemia were randomly assigned at a 1:1 ratio to receive either hybutimibe (20 mg/d) plus atorvastatin (10 mg/d) once daily or atorvastatin (20 mg/d) for 12 consecutive weeks. For patients assigned to treatment group, the following drugs were prohibited during the whole study period: fibrates, niacins, any other lipid-lowering drugs or drugs that may affect blood lipid levels, cyclosporine, digoxin, and anticoagulants. Necessary symptomatic treatments were provided if adverse reactions or acute symptoms/signs occurred.

**Figure 1:**
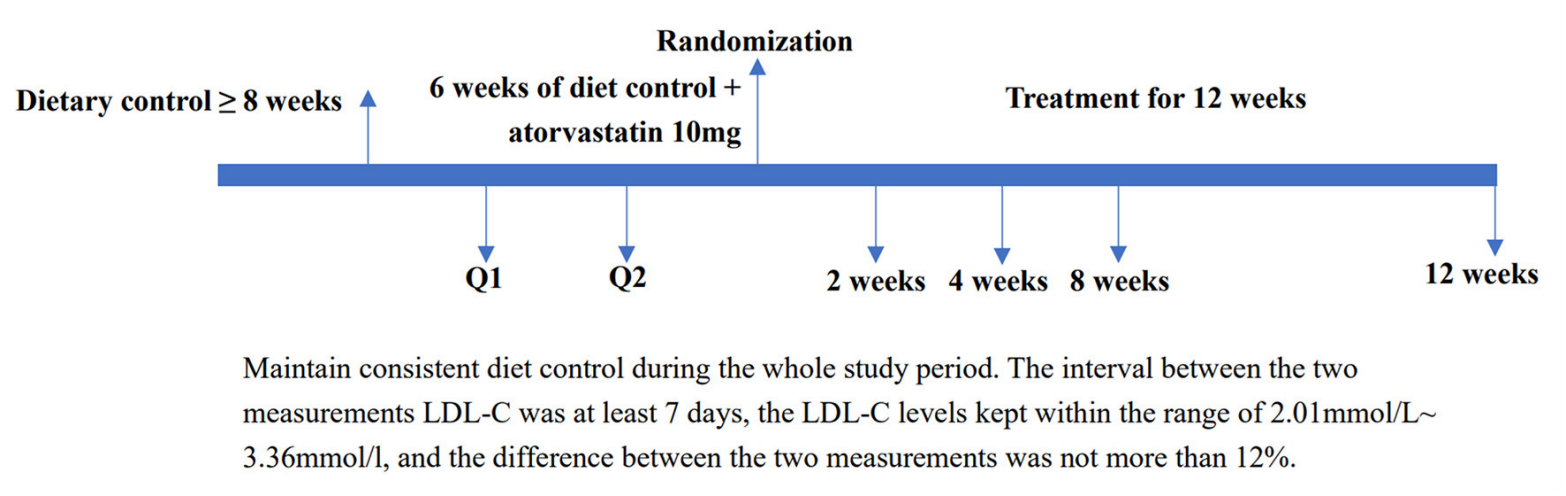
Study process from screening, randomization assignment, treatment to end of study. LDL-C, low-density lipoprotein cholesterol.

Randomization through the Interactive Web Response System was performed by the Department of Biostatistics of Nanjing Medical University. Study participants, investigators, and research staff were blinded to the assignment of the patients to treatment groups. Sponsors or designees involved in the study design and data analysis were also blinded to this assignment until the independent data monitoring committee recommended stopping the study. The detailed study process from screening, randomization assignment, treatment to end of study was shown in Figure 1.

### Outcomes and assessments

The primary outcome of this study was the rate of change of plasma LDL-C level at 12 weeks from baseline, which was calculated as follows: (plasma LDL-C level at 12 weeks–LDL-C level at baseline)/LDL-C level at baseline. The secondary outcomes were rates of change of plasma total cholesterol (TC), triglyceride (TG), HDL-C, non-HDL-C, apoprotein B (Apo B), and apoprotein A1 (Apo-A1) levels at 2, 4, 8, and 12 weeks from baseline and rates of change of plasma LDL-C level at 2, 4, and 8 weeks from baseline. Blood lipid levels were measured by the central laboratory, an independent third-party laboratory: Kunhao Ruicheng Laboratory (Q2 Solutions), according to the standard operation protocol and quality control criteria. The research nurses in each center collected blood samples, and then stored and transport them to the central laboratory in batches on a regular basis in accordance with the sample operation manual issued by the central laboratory test. Safety was assessed and recorded, including adverse effects (graded according to the National Cancer Institute Common Terminology Criteria for Adverse Events Version 4.03) by investigators blindly, laboratory measurements, and other safety data, from the beginning of the study treatment to 12 weeks later.

### Statistical analyses

This study was designed to test the null hypothesis that the rate of change of LDL-C level at 12 weeks from baseline was greater in the group treated with hybutimibe plus atorvastatin than in the group treated with placebo plus atorvastatin. A total of 120 patients in each of the two groups were needed for the study to have statistical power of 90% to detect a clinically meaningful difference in LDL-C improvement rate of 8% between the two groups (12), if tested at a one-sided significance level of α = 0.025 and with a drop-out rate of 20%.

We calculated the mean, standard deviation, median, minimum, and maximum for continuous variables and frequency and percentage for categorical variables. Rates of change of LDL-C at 12 weeks from baseline were compared between the two treatment groups by analysis of covariance with the dependent variable of rate of change of LDL-C and the covariate of LDL-C at baseline. To estimate the difference in rates of change between the two groups (hybutimibe plus atorvastatin vs. atorvastatin), least squares means and 95% confidence interval (CI) for the rate of change of LDL-C were calculated. If the lower limit of the 95% CI of the estimated difference between the two groups was <0, hybutimibe plus atorvastatin therapy was considered to be better than atorvastatin monotherapy. Subgroup analyses of primary outcome stratified by location, age (<60 and ≥60 years), sex (male and female), history of coronary heart disease (yes and no), history of stroke (yes and no), and diabetes (yes and no) were also conducted. In addition, sensitivity analysis was applied to exclude the data from the First Affiliated Hospital of Hebei Medical University due to its poor quality. Analysis of variance was used to compare the rates of change of LDL-C at 2, 4, and 8 weeks and the rates of change of non-HDL-C, HDL-C, TC, TG, Apo B, and Apo-A1 levels at 2, 4, 8, and 12 weeks from baseline between the two groups. The frequency and rate of emerging treatment-related adverse events were reported.

Intention-to-treat analyses compared the baseline characteristics of groups defined by the randomization procedure and included all participants irrespective of adherence to the assigned intervention or to the protocol. The modified intention-to-treat (mITT) population (including patients who received at least one dose of the study drugs) was used for the primary and secondary outcomes. Last observation carried forward was used to impute missing values of the primary outcome. The per-protocol population (PPS) which included all patients completing the study without major protocol deviations was used for supplemental analyses of the primary and secondary outcomes. The safety analyses included all patients who received at least one dose of the study drugs.

All statistical analyses were conducted using SAS software (version 9.4; SAS Institute). Two-sided *P* < 0.05 were considered statistically significant.

## Results

**Figure 2:**
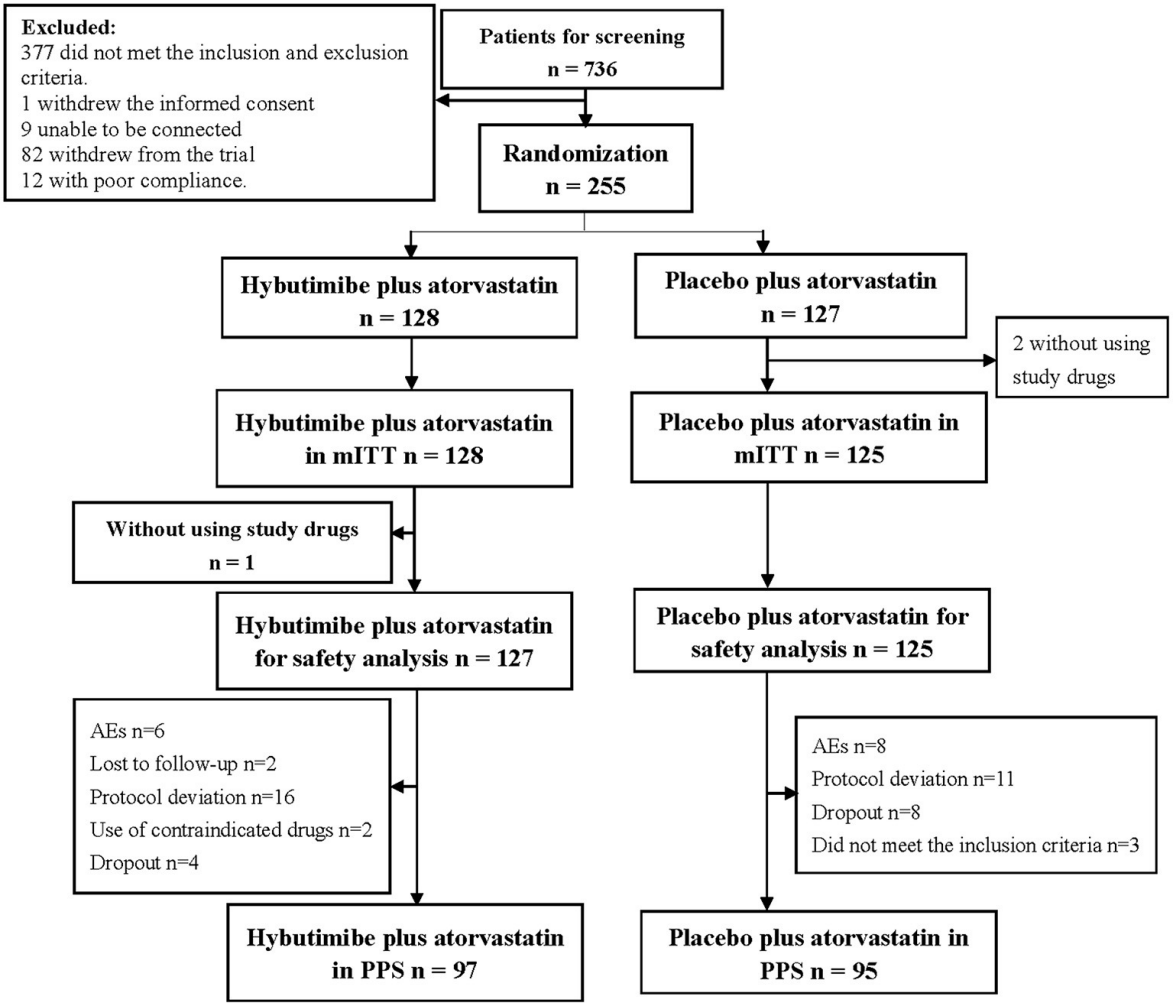
Diagram for patients' selection. mITT, modified intention-to-treat; AEs, adverse events; PPS, per-protocol set.

From April 2016 to January 2018, a total of 736 patients were screened from 23 centers in China, among whom 377 cases were excluded according to the inclusion and exclusion criteria, 1 case withdrew informed consent, 9 cases were lost to follow-up, 82 withdrew from the trial, and 12 had poor compliance. Thus, finally 255 patients were enrolled in this study: 128 in the hybutimibe plus atorvastatin treatment group and 125 in the atorvastatin treatment group (Figure 2). Among these 255 patients, 2 who did not use the study drugs in the atorvastatin treatment group were excluded from mITT analysis.

A total of 62.06% of the patients were men, while 70.36% had coronary heart disease, 20.16% had suffered stroke, and 46.25% had diabetes. TC, TG, HDL-C, LDL-C, Apo-A1, and Apo B levels at baseline were comparable in the hybutimibe plus atorvastatin and atorvastatin treatment groups (Table 1).

**Table 1:** Baseline characteristics of the modified intention-to-treat population.

**Characteristics**	**Hybutimibe plus atorvastatin (*n* = 128)**	**Atorvastatin (*n* = 125)**	***P***
Age, years	62.12 ± 6.37	60.89 ± 7.79	0.1714
Sex, Males	79 (61.72)	78 (62.40)	0.9111
BMI, kg/m^2^	25.24 ± 3.21	25.63 ± 3.09	0.3278
Comorbidity			0.5774
Coronary heart disease	90 (70.31)	88 (70.40)	
Stroke	30 (23.44)	21 (16.80)	
Diabetes	62 (48.44)	55 (44.00)	
SBP, mmHg	127.73 ± 11.66	127.72 ± 10.73	0.9919
DBP, mmHg	76.40 ± 8.52	77.74 ± 8.28	0.2066
Heart rate, times per min	71.97 ± 8.68	72.42 ± 9.05	0.6886
Respiratory, times per min	18.09 ± 1.33	18.01 ± 1.54	0.6668
TC, mmol/L	4.21 ± 0.45	4.20 ± 0.48	0.8317
TG, mmol/L	1.59 ± 0.61	1.61 ± 0.73	0.8017
HDL-C, mmol/L	1.24 ± 0.27	1.29 ± 0.31	0.1478
LDL-C, mmol/L	2.61 ± 0.30	2.58 ± 0.31	0.3938
APO-A1, g/L	1.37 ± 0.21	1.42 ± 0.23	0.1102
APO-B, g/L	0.89 ± 0.11	0.90 ± 0.11	0.9329

BMI, body mass index; SBP, systolic blood pressure; DBP, diastolic blood pressure; TC, total cholesterol; TG, triglyceride; HDL-C, high-density lipoprotein cholesterol; LDL-C, low-density lipoprotein cholesterol; APO-A1, apoprotein A1; APO-B, apoprotein B.

After 12 weeks of treatment, the LDL-C levels changed from 2.61 mmol/L (±0.30) at baseline to 2.18 mmol/L (±0.45) in the hybutimibe plus atorvastatin group and from 2.58 (±0.31) to 2.40 (±0.46) mmol/L in the atorvastatin group (*P* < 0.0001), in mITT analysis (Table 2). The rate of change in the hybutimibe plus atorvastatin treatment group was significantly higher than that in the atorvastatin treatment group (*P* < 0.0001); the estimated mean rates of change were −16.39 (95% CI: −19.04, −13.74) and −6.75 (−9.48, −4.02), respectively. We further adjusted for LDL-C level at baseline or LDL-C at baseline, age, and body mass index (BMI), obtaining consistent results (all *P* < 0.0001). Consistent with this, for PPS analysis, the LDL-C levels were significantly improved for both groups after treatment (all *P* < 0.0001), and the rate of change in the hybutimibe plus atorvastatin treatment group was significantly higher than that in the atorvastatin treatment group, even after adjusting for LDL-C level at baseline or LDL-C at baseline, age, and BMI (range of *P*-values: 0.0002–0.0007).

**Table 2:** Change rates of LDL-C levels at 12 weeks from baseline in two treatment groups.

**LDL-C changes**		**Hybutimibe plus atorvastatin (*n* = 128)**	**Atorvastatin (*n* = 125)**	***P*-value**
mITT	Pre-treatment, mmol/L	2.61 ± 0.30	2.58 ± 0.31	0.3938
	12 weeks, mmol/L	2.18 ± 0.45	2.40 ± 0.46	0.0001
	Changes *P*-value	<0.0001	<0.0001	
	Rate of changes (95% CI)	−16.39 (−19.04, −13.74)	−6.75 (−9.48, −4.02)	<0.0001
	Adjusted rate of changes (95% CI)^*^	−16.33 (−18.99, −13.67)	−6.82 (−9.51, −4.13)	<0.0001
	Adjusted rate of changes, (95% CI)^#^	−16.24 (−18.94, −13.54)	−6.80 (−9.51, −4.08)	<0.0001
PPS	Pre-treatment, mmol/L	2.64 ± 0.30	2.55 ± 0.30	0.0557
	12 weeks, mmol/L	2.18 ± 0.44	2.32 ± 0.41	0.0183
	Changes *P*-value	<0.0001	<0.0001	
	Rate of changes (95% CI)	−17.21 (−20.39, −14.02)	−8.86 (−11.79, −5.93)	0.0002
	Adjusted rate of changes (95% CI)^*^	−16.87 (−19.87, −13.86)	−9.21 (−12.24, −6.17)	0.0005
	Adjusted rate of changes (95% CI)^#^	−16.83 (−19.91, −13.75)	−9.12 (−12.20, −6.04)	0.0007

* Adjusted for LDL-C levels at baseline.

#Adjusted for LDL-C at baseline, age, and BMI.

LDL-C, low-density lipoprotein cholesterol; mITT, modified intention-to-treat; CI, confidential interval; PPS, per-protocol set; BMI, body mass index.

The rates of change of LDL-C in subgroup analyses stratified by age, sex, history of stroke, and history of diabetes showed a consistent improvement upon treatment with hybutimibe plus atorvastatin, compared with the status upon atorvastatin treatment (Table 3). However, in patients without coronary heart disease, the rate of change of LDL-C was improved upon treatment with hybutimibe plus atorvastatin [−13.14 (95% CI: −18.69, −7.59) vs. −5.87 (95% CI: −12.20, 0.46)], but this was not statistically significant (*P* = 0.0835).

**Table 3:** Subgroup analyses of rates of changes in LDL-C at 12 weeks from baseline.

**Subgroups**		**Hybutimibe plus atorvastatin, mmol/l**	**Atorvastatin, mmol/l**	***P*-value**
Age	<60 yrs	−15.11 (−20.74, −9.48) (*n* = 38)	−7.91 (−11.90, −3.93) (*n* = 50)	0.0331
	≥60 yrs	−16.93 (−19.93, −13.94) (*n* = 90)	−5.97 (−9.73, −2.22) (*n* = 75)	<0.0001
Sex	Males	−16.43 (−19.67, −13.18) (*n* = 79)	−7.55 (−10.79, −4.30) (*n* = 78)	0.0002
	Females	−16.34 (−21.03, −11.65) (*n* = 49)	−5.42 (−10.43, −0.42) (*n* = 47)	0.0018
Coronary heart disease	Yes	−17.77 (−20.75, −14.78) (*n* = 90)	−7.12 (−10.03, −4.20) (*n* = 88)	<0.0001
	No	−13.14 (−18.69, −7.59) (*n* = 38)	−5.87 (−12.20, 0.46) (*n* = 37)	0.0835
Stroke	Yes	−16.05 (−21.06, −11.05) (*n* = 30)	−3.64 (−12.56, 5.28) (*n* = 21)	0.0098
	No	−16.50 (−19.65, −13.35) (*n* = 98)	−7.38 (−10.19, −4.57) (*n* = 104)	<0.0001
Diabetes	Yes	−16.10 (−19.84, −12.36) (*n* = 62)	−6.30 (−10.71, −1.89) (*n* = 55)	0.0009
	No	−16.67 (−20.52, −12.82) (*n* = 66)	−7.10 (−10.63, −3.58) (*n* = 70)	0.0004

**Figure 3:**
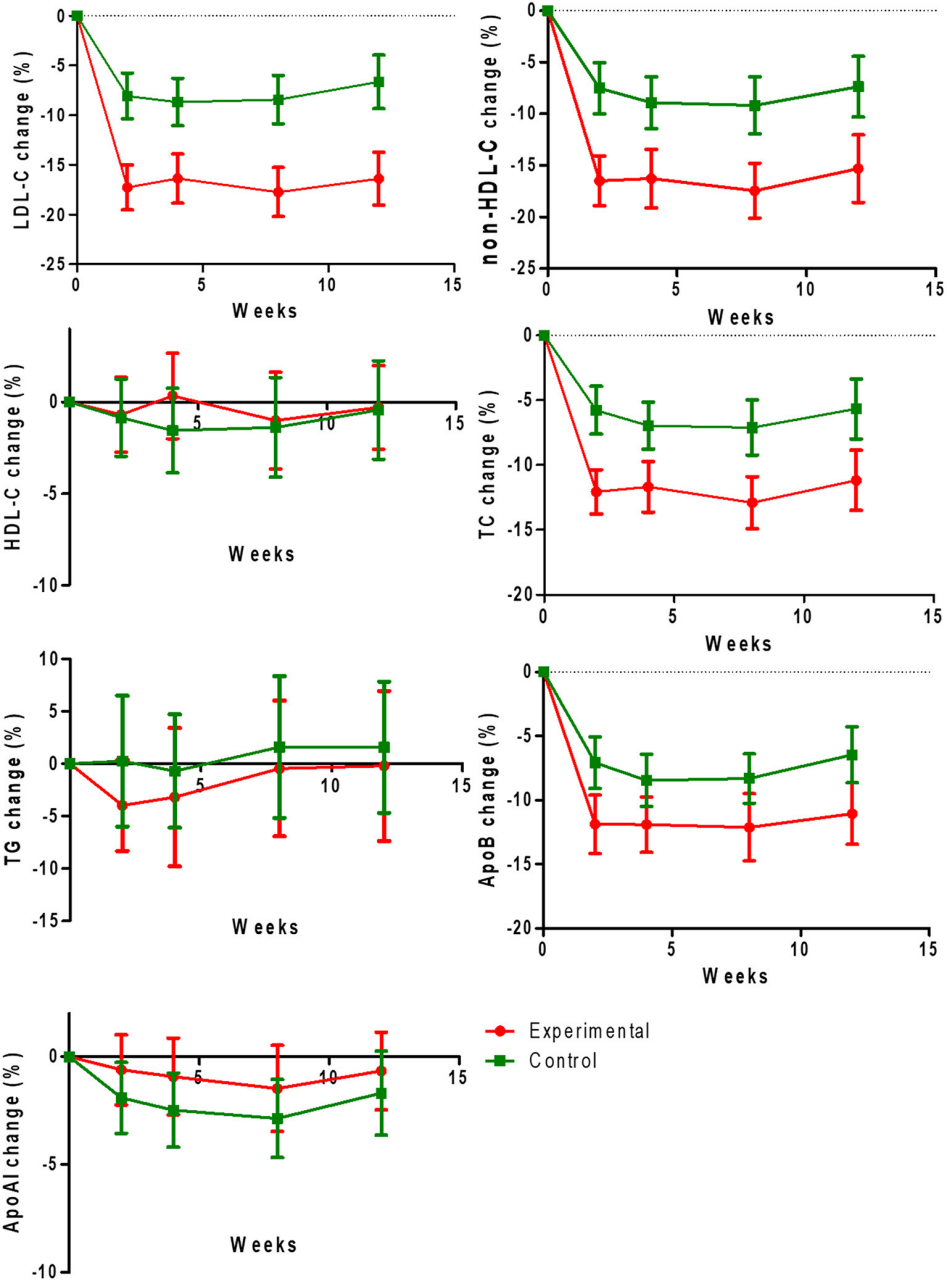
Change rates of low-density lipoprotein cholesterol (LDL-C), non-HDL-C (high-density lipoprotein cholesterol), HDL-C, TC (total cholesterol), TG (triglyceride), apoprotein B (Apo-B), and apoprotein A1 (Apo-A1) levels at 12 weeks from baseline in two treatment groups.

Regarding the secondary outcomes, we found significant decreases of the rates of change of non-HDL-C (−16.49 ± 13.43 vs. −7.52 ± 13.59%, −16.26 ± 15.82 vs. −8.91 ± 13.51%, −17.45 ± 14.38 vs. −9.17 ± 14.82%, −15.30 ± 17.84 vs. −7.36 ± 15.74%), TC (−12.06 ± 9.57 vs. −5.77 ± 10.12%, −11.67 ± 10.89 vs. −6.96 ± 9.73%, −12.90 ± 10.84 vs. −7.11 ± 11.38%, −11.18 ± 12.67 vs. −5.66 ± 12.29%), and Apo B (−11.88 ± 12.80 vs. −7.07 ± 11.15%, −11.91 ± 12.04 vs. −8.46 ± 11.00%, −12.12 ± 14.25 vs. −8.31 ± 10.33%, −11.05 ± 13.15 vs. −6.46 ± 11.67%) at 2, 4, 8, and 12 weeks (all *P* < 0.05), but non-significant differences were observed for HDL-C, TG, and Apo-A1 (all *P* > 0.05; Figures 3A–G).

During the study period, a total of 65 of 128 patients in the hybutimibe plus atorvastatin group and 77 of 125 patients in the atorvastatin group occurred adverse events (AEs; Table 4). The primary AEs were upper respiratory tract infection, dizziness, and abdominal discomfort. AEs with an incidence of ≥5% included upper respiratory tract infection [16 (12.60%) vs. 19 (15.20%)], dizziness [7 (5.51%) vs. 5 (4.00%)], and abdominal discomfort [8 (6.30%) vs. 4 (3.20%)]. Among them, 17 vs. 18 were drug-related AEs, and 3 vs. 6 were drug-related AEs leading to suspension of the trial, 8 vs. 9 were serious AEs, 6 vs. 12 were AEs leading to suspension of the trial, 0 vs. 1 were serious AEs related to the study drugs, and there were no AEs or drug-related AEs leading to death.

**Table 4:** Adverse events.

**Adverse events**	**Hybutimibe plus atorvastatin (*****n*** = **127)**		**Atorvastatin (*****n*** = **125)**	
	***n***	**Patients (%)**	***n***	**Patients (%)**
Total	173	65 (51.18)	159	77 (61.60)
Adverse events leading to suspension of the trial	6	5 (3.94)	12	10 (8.00)
Serious adverse events	8	7 (5.51)	9	9 (7.20)
Adverse events resulting in death	0	0 (0.00)	0	0 (0.00)
Drug-related adverse events	17	10 (7.87)	18	17 (13.60)
Drug-related adverse events that led to the suspension of the trial	3	2 (1.57)	6	6 (4.80)
Serious adverse events related with study drugs	0	0 (0.00)	1	1 (0.80)
Drug-related adverse events leading to death	0	0 (0.00)	0	0 (0.00)

We applied a sensitivity analysis included the patients from the First Affiliated Hospital of Hebei Medical University and a consistent main result was concluded. For mITT (5 in treatment and 3 in control group), after 12-week treatment, the change rate of LDL-C from baseline was −17.64 ± 12.87% in treatment group, while it was −7.51± 14.62% in control group, and the difference between two groups was statistically significant (*P* < 0.0001). The difference between two groups was −10.13% (95% CI: −13.60%, −6.67%). For PPS (5 in treatment and 2 in control group), similar results were found in PPS. After 12-week therapy, LDL-C change rate from baseline was −18.92 ± 12.67% in treatment group, and it was −9.57 ± 13.41% in control group, also the between group difference was −9.35% (95%CI: −13.14%, −5.57%, *P* < 0.0001).

## Discussion

This randomized, double-blind, paralleled, multicenter phase III study found that hybutimibe plus atorvastatin significantly improved the LDL-C level in hypercholesterolemia patients with atherosclerotic cardiovascular disease and/or “equal-risk diseases” after 12 weeks of treatment, with a mean rate of change of −16.39, compared with atorvastatin monotherapy, for which the mean rate of change was −6.75. Additionally, hybutimibe plus atorvastatin significantly improved non-HDL-C, TC, and Apo B, but had non-significant effects on HDL-C, TG, and ApoA1 levels. The subgroup and sensitivity analyses did not show any inconsistent results and the safety analysis showed that a well tolerability.

We showed that the combination of hybutimibe plus atorvastatin is associated with significant improvements in LDL-C (−16.39%), non-HDL-C (−15.30%), TC (−11.18%), and Apo B (−11.05%) levels in patients with atherosclerotic cardiovascular disease and risk equivalents, although clinical benefits on HDL-C, TG, and Apo-A1 were not observed compared with atorvastatin monotherapy. The efficacy of hybutimibe plus atorvastatin was noted, irrespective of previous therapy, age, and a history of coronary heart disease, stroke, or diabetes. A previous double-blind study included 628 patients with baseline LDL-C of 145–250 mg/dL (3.756–6.475 mmol/L) and triglycerides of ≤350 mg/dL (3.955 mmol/l) who were randomly assigned to receive one of the following for 12 weeks: ezetimibe (10 mg/d); atorvastatin (10, 20, 40, or 80 mg/d); ezetimibe (10 mg) plus atorvastatin (10, 20, 40, or 80 mg/d); or placebo (16). This study found that the co-administration of ezetimibe provided a significant additional 12% reduction of LDL-C, 3% increase of HDL-C, 8% reduction of triglyceride, and 10% reduction of high sensitivity C-reactive protein vs. atorvastatin alone (16). Ezetimibe plus atorvastatin provided LDL-C reductions of 50–60%, TC reductions of 30–40%, and HDL-C increases of 5–9%, depending on the dose of atorvastatin. Although we did not make a head-to-head comparison of ezetimibe and hybutimibe in this study, we applied several comparisons in pharmacokinetic and efficacy since these two drugs share a similar mechanism in lowering lipids. Compared with ezetimibe, hybutimibe showed a smaller effect on LDL-C reduction, however, the treatment duration of this study was only 12 weeks, and our study included the patients with a wide age range of 18–75 years, thus it is difficult to identify which one is more efficient. The chemical construction changes in hybutimibe make it easier to bind to glucuronic acid, which led to more excretion of hybutimibe (15.6% of dose) than ezetimibe (11% of dose) in urine (11). Additionally, the peak time of hybutimibe is 0.5–12 h, while it is 4–12 h for ezetimibe, which means hybutimibe can quickly reach the treatment levels and play its drug efficacy faster in human body (12). As we know, these current studies are large different in study design, such as inclusion and exclusion criteria of participants, treatment duration and dosage, sample size, and ethnicity difference in participants, which result in substantially differences in lipid-lowering effects, thus the comparison across the studies should be more cautiously. Taken all these together, hybutimibe might be a potential efficacy drug for lipid lowering, although its efficacy and safety warrant further large scaled and long-duration study to verify.

It has been reported that using ezetimibe together with atorvastatin can increase the risk of side effects such as liver damage and a rare but serious condition called rhabdomyolysis, which involves the breakdown of skeletal muscle tissue (9, 17). In some cases, rhabdomyolysis can cause kidney damage (18) and even death (19, 20). Rhabdomyolysis was not observed in either group in the present study, although five patients in the combination therapy group and seven in the monotherapy group reported elevated creatinine kinase (CK) levels. Five AEs in the monotherapy group and only two in the combination therapy group were related to the study drugs. Only two cases in the combined treatment group reported a lack of elevation of CK levels to ≥10 times the upper limit of the normal range. In summary, our phase III study suggested that the combination of hybutimibe and atorvastatin does not cause additional side effects and has acceptable safety and tolerability. Since this study was only performed for a short period, had a small sample size, and only included Chinese patients, there is a need for further assessment of long-term and rare side effects, especially regarding the interactions with food and drugs.

All patients included in this study had combination with atherosclerotic cardiovascular disease and or “equal-risk diseases,” and also did not achieve the target blood LDL-C level after treatment with 10 mg of atorvastatin for at least 6 weeks. These patients are generally at high or very high risk of atherosclerotic cardiovascular diseases (1). As we know, whether identifying the high and very-high-risk atherosclerotic cardiovascular disease patients is disputable (21). In a clinical context, the identification of high-risk patients is important in order to match patients with particular treatment intensities. Although evidence reported that long-term and high-intensity statin treatment might introduce a series of side effects, such as neurological effects, gastrointestinal hemorrhage, muscle pain and damage, liver damage, increased blood glucose, and a risk of type 2 diabetes (22), Statins are the most thoroughly studied and time-tested treatment for cardiovascular prevention and treatment and are very well-tolerated by the vast majority of users, with rates of serious complications that are exceeding rare (23). A study including 27,775 patients found that the “very high-risk” patients defined in accordance with the 2018 AHA/ACC/Multisociety guidelines had nearly three times higher risk of developing future ASCVD events than those without a very high risk (24). This study suggested that it is important to identify populations with a very high risk of atherosclerotic cardiovascular diseases and that these patients should undergo intensive lipid-lowering therapy (1).

A possible limitation of this study is that the sample size was small, reducing the statistical power in several subgroup analyses and when exploring the heterogeneity of hybutimibe plus atorvastatin combination therapy for lipid control. In addition, all patients in this study were Chinese, so care should be taken when extrapolating the current conclusion to other populations. Moreover, the efficacy and safety in other ethnic groups need further evaluation. We used the mITT to evaluate the primary outcome which is slightly different from the ITT analysis, because we considered 2 patients without use any study drugs. Finally, this was a short-term follow-up study, so a study with a large sample size is needed to confirm the longer-term efficacy and safety, especially for atherosclerotic cardiovascular outcomes.

## Data availability statement

The raw data supporting the conclusions of this article will be made available by the authors, without undue reservation.

## Ethics statement

The studies involving human participants were reviewed and approved by Ethics Committee of the First Hospital of Peking University [No. (2015) Drug Registration No. (56)]. The patients/participants provided their written informed consent to participate in this study.

## Author contributions

LQ: conceptualization. JC, XL, XQ, and CD: data collection. XC, XGu, WX, and SZ: statistical analysis. YD, MZ, KH, and LT: data interpretation and supervision. XGuo, FW, GF, and JL: visualization and project administration. YH: writing and editing of the manuscript. All authors have read and approved the final manuscript.

## Funding

This study was supported by National science and technology major projects-new drugs creation and development [Grant Number 2013ZX09402101].

## Conflict of interest

The authors declare that the research was conducted in the absence of any commercial or financial relationships that could be construed as a potential conflict of interest.

## Publisher's note

All claims expressed in this article are solely those of the authors and do not necessarily represent those of their affiliated organizations, or those of the publisher, the editors and the reviewers. Any product that may be evaluated in this article, or claim that may be made by its manufacturer, is not guaranteed or endorsed by the publisher.
